# IL-1 Coordinates the Neutrophil Response to *C*. *albicans* in the Oral Mucosa

**DOI:** 10.1371/journal.ppat.1005882

**Published:** 2016-09-15

**Authors:** Simon Altmeier, Albulena Toska, Florian Sparber, Alvaro Teijeira, Cornelia Halin, Salomé LeibundGut-Landmann

**Affiliations:** 1 Section of Immunology, Vetsuisse Faculty, University of Zürich, Zürich, Switzerland; 2 Institute of Pharmaceutical Sciences, Swiss Federal Institute of Technology, Zürich, Switzerland; University of Birmingham, UNITED KINGDOM

## Abstract

Mucosal infections with *Candida albicans* belong to the most frequent forms of fungal diseases. Host protection is conferred by cellular immunity; however, the induction of antifungal immunity is not well understood. Using a mouse model of oropharyngeal candidiasis (OPC) we show that interleukin-1 receptor (IL-1R) signaling is critical for fungal control at the onset of infection through its impact on neutrophils at two levels. We demonstrate that both the recruitment of circulating neutrophils to the site of infection and the mobilization of newly generated neutrophils from the bone marrow depended on IL-1R. Consistently, IL-1R-deficient mice displayed impaired chemokine production at the site of infection and defective secretion of granulocyte colony-stimulating factor (G-CSF) in the circulation in response to *C*. *albicans*. Strikingly, endothelial cells were identified as the primary cellular source of G-CSF during OPC, which responded to IL-1α that was released from keratinocytes in the infected tissue. The IL-1-dependent crosstalk between two different cellular subsets of the nonhematopoietic compartment was confirmed *in vitro* using a novel murine tongue-derived keratinocyte cell line and an established endothelial cell line. These data establish a new link between IL-1 and granulopoiesis in the context of fungal infection. Together, we identified two complementary mechanisms coordinating the neutrophil response in the oral mucosa, which is critical for preventing fungal growth and dissemination, and thus protects the host from disease.

## Author Summary

The opportunistic pathogen *Candida albicans* is a major risk factor for immunosuppressed individuals, and oropharyngeal candidiasis (OPC) is a frequent complication in patients with weakened cellular immunity. The cytokine interleukin-17 (IL-17) plays a critical role for antifungal host defense and was proposed to act by regulating neutrophil recruitment to the oral mucosa. However, although IL-17 can promote neutrophil trafficking in some situations, we recently showed in a mouse model that this is not the case during OPC. Thus, the mechanism governing the neutrophil response to *C*. *albicans* remained to be determined. Here, we demonstrate an essential role of IL-1 receptor (IL-1R) signaling in the recruitment of neutrophils from the circulation to the infected tissue via enhanced secretion of chemokines and increased output of neutrophils from the bone marrow. We found that IL-1α is released from keratinocytes upon invasion of C. *albicans* and acts on endothelial cells to induce the production of granulocyte colony-stimulating factor (G-CSF), a key trigger of emergency granulopoiesis. Thereby, IL-1R signaling translates the local response to the fungus in the oral mucosa into a systemic response that critically contributes to protection from infection.

## Introduction

The opportunistic fungal pathogen *Candida albicans* has emerged as a significant cause of morbidity and mortality worldwide, particularly in immunocompromised individuals [1]. Of the diverse forms of disease manifestations, mucosal infections with *C*. *albicans* are by far most abundant [2]. The symptoms reach from mild forms of infection to chronic or recurrent diseases. No licensed fungal vaccines are currently available to prevent disease, and toxicity and resistance to available drugs compromise the effective management of patients. With the ever-increasing population of immunocompromised patients, *C*. *albicans* infections thus represent an important socio-economic challenge worldwide.

The epithelium constitutes the first point of contact between the fungus and the host [3]. It provides an important physical barrier to prevent fungal invasion. Moreover, it has the capacity to sense and respond to the fungus. By producing inflammatory mediators and antifungal defense molecules the epithelium actively participates in the host response and together with leukocytes, including neutrophils and IL-17-producing lymphocytes, contributes to limiting fungal (over)growth. Diverse mutual interactions between leukocytes and the epithelium are critical for mounting a broadly protective response against *C*. *albicans*. The epithelium elicits signals in response to the fungus that can promote the inflammatory response [3], while cytokines such as IL-17, produced by leukocytes, act on the epithelium to enhance its barrier function and antimicrobial activity [4,5]. Neutrophils have been shown to rapidly accumulate in the oral mucosa in response to *C*. *albicans* infection, and they critically contribute to prevent invasion of the fungus in underlying tissues and dissemination to the circulation and visceral organs as was shown in a model of acute oropharyngeal candidiasis (OPC) [6,7]. The relevance of neutrophils in protection from oropharyngeal candidiasis is also evidenced by the high incidence of the disease in hemato-oncological patients with bone marrow aplasia [8,9].

Neutrophils comprise a major proportion of circulating peripheral blood leukocytes. They are generated from granulocyte-macrophage progenitors in the bone marrow under the control of granulopoietic growth factors, primarily granulocyte colony-stimulating factor (G-CSF) [10]. During acute infection, granulopoiesis is massively enhanced to comply with the increased demand for neutrophils in host defense [11]. Control mechanisms of this demand-adapted hematopoiesis involve long-distance regulatory feedback loops induced at the site of infection where neutrophils act, which is usually distant from the production site of neutrophils in the bone marrow. Increased release of G-CSF in response to infectious and/or inflammatory insult plays a key role in this process [11]. Given the potentially harmful effects of dysregulated neutrophils, granulopoiesis and neutrophil trafficking is under tight control and regulated in a tissue-specific manner [12].

With the discovery of interleukin-17 (IL-17) and the realization of its critical role in defense against mucocutaneous candidiasis [5,13], it was postulated that IL-17 mediates protection by promoting the neutrophil response. Indeed, IL-17 signaling can enhance expression of neutrophil cytopoietic and chemotactic factors in response to *C*. *albicans* [14]. However, we recently demonstrated that neutrophils are recruited normally to the site of infection in IL-17 receptor-deficient mice, thus that the IL-17 pathway is not required for the neutrophil response during OPC [6]. Therefore, although neutrophil trafficking may be regulated by IL-17 in some tissues and in response to certain pathogens [15–19]—this is not the case during *C*. *albicans* infection in the oral mucosa.

Alternative candidate factors regulating the neutrophil response include IL-1. In fact, secretion of both IL-1α and IL-1β are efficiently induced in dendritic cells and macrophages when stimulated with *C*. *albicans* [20–23] and both IL-1 family members were shown to contribute to protection from systemic infection [24]. Epithelial cells also secrete IL-1 cytokines when triggered with *C*. *albicans* [25–27], although in mice, unlike in humans, keratinocytes produce only IL-1α but no IL-1β [28]. Whether and how IL-1 cytokines contribute to antifungal defense in barrier tissues remains poorly defined.

Using a mouse model of oropharyngeal candidiasis, we found that IL-1R signaling is critical for host defense by regulating the neutrophil response. We show here that IL-1 acts by two complementary mechanisms. First, it regulates the production of neutrophil-chemotactic factors by epithelial cells for the recruitment of neutrophils from the circulating pool to the site of infection. Second, it induces G-CSF production by the endothelium for enhanced neutrophil output from the bone marrow to meet the increased demand in response to infection. Release of IL-1α from keratinocytes upon contact with *C*. *albicans* is critical for mediating this crosstalk between the epithelium and the endothelium in the oral mucosa.

## Results

### IL-1 receptor signaling makes an important contribution to neutrophil recruitment to the oral mucosa

**Fig 1:**
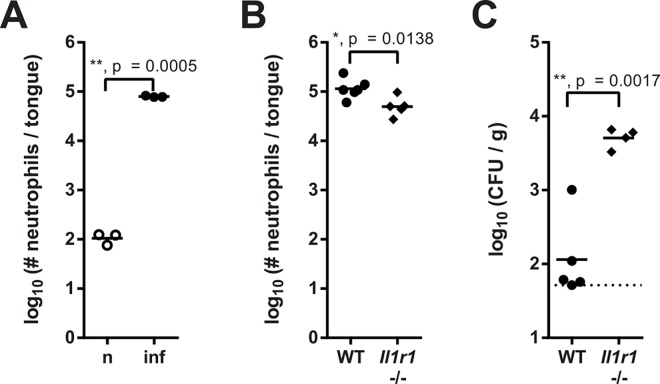
IL-1R signaling makes an important contribution to neutrophil recruitment to the oral mucosa. (**A**) CD45^+^ Ly6C^int^ Ly6G^+^ neutrophils were quantified by flow cytometry in the tongues of naïve (n) and *C*. *albicans* infected (inf) WT mice 24 hours post-infection. (**B**) Neutrophils were quantified in the tongues of infected WT and *Il1r1* ^-/-^ mice 24 hours post-infection. (**C**) The fungal burden in the tongues of infected WT and *Il1r1* ^-/-^ mice was determined on day 3 post-infection. Each symbol represents an individual mouse, and the lines represent the geometric mean of each group. The dotted line represents the detection limit. Data are pooled from two (B) or representative of three (A) or two (C) independent experiments. Statistical analysis was performed using log_10_ transformation and Student’s t-test with Welch’s correction.

Infection of mice with *C*. *albicans* via the oropharyngeal route induces a rapid inflammatory response, characterized by a massive recruitment of neutrophils to the oral mucosa (**Fig 1A, S1 Fig**) [6,7,29], which accumulate in proximity to where *C*. *albicans* hyphae invade the keratinocyte barrier on the dorsal and ventral side of the tongue [6]. Following from our previous findings that neutrophil trafficking during OPC was independent of the IL-17 pathway [6], we sought after factors responsible for controlling neutrophil recruitment in response to *C*. *albicans*. We found that mice lacking the IL-1 receptor (IL-1R) recruited significantly less neutrophils to the site of infection than their wild type (WT) counterparts (**Fig 1B**). In consequence *Il1r1* ^-/-^ mice were unable to control the fungus and displayed an increased fungal load in the tongue on day 3 post-infection (**Fig 1C**). These data indicated clearly that IL-1R signaling was critical for the neutrophil response during OPC.

### The induction of neutrophil-recruiting chemokines in the oral mucosa depends on IL-1R signaling

**Fig 2:**
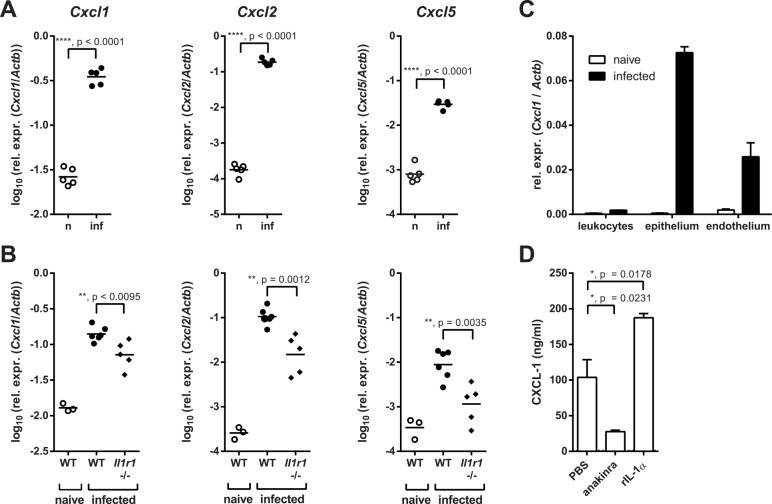
Neutrophil-recruiting chemokines in the oral mucosa are reduced in absence of IL-1R signaling. (**A**) Chemokine expression in the tongues of naïve (n) and infected (inf) WT mice was measured by qRT-PCR 24 hours post-infection. (**B**) Chemokine expression in the tongues of naïve and infected WT and infected *Il1r1* ^-/-^ mice 24 hours post-infection. (**C**) CD45^+^ leukocytes, CD45^-^ EpCAM^+^ CD31^-^ epithelial cells, and CD45^-^ EpCAM^-^ CD31^+^ endothelial cells were isolated from the tongues of naïve and infected WT mice by FACS sorting 24 hours post-infection, and *Cxcl1* mRNA was quantified by qRT-PCR. (**D**) The tongue-derived keratinocyte (TDK) cell line was treated with recombinant IL-1α (20 ng/ml), anakinra (250 μg/ml), or PBS, and CXCL1 levels in the culture supernatant were determined by ELISA. Bar graphs show the group mean + SD. Data are representative of two (A, C–D) or pooled from two (B) independent experiments, with the exception of the naïve group in B, which is from one experiment. Statistical analysis was performed using log_10_ transformation and Student’s t-test with Welch’s correction (A) or one-way ANOVA with Dunnett’s test (B, D).

The rapid accumulation of neutrophils in the oral mucosa of infected mice was paralleled by a strong induction of the neutrophil-recruiting chemokines CXCL1, CXCL2 and CXCL5 (**Fig 2A**). Consistent with the role of IL-1 in neutrophil recruitment, chemokine expression in the oral epithelium was impaired in absence of IL-1R signaling (**Fig 2B**), while basal levels were comparable (**S2 Fig**). To determine the cellular compartment responsible for chemokine production, we sorted cellular subsets from the tongue of naïve and *C*. *albicans*-infected mice on day 1 post-infection, the peak of the neutrophil response, including CD45^+^ leukocytes, CD45^-^ EpCAM^+^ CD31^-^ keratinocytes and CD45^-^ EpCAM^-^ CD31^+^ endothelial cells (**S3 Fig**) and analyzed chemokine expression at the transcriptional level. The keratinocyte fraction displayed the highest RNA levels, and the induction in response to infection was most prominent in this population (**Fig 2C**). Consistent with this result, a cell line of tongue-derived keratinocytes (TDKs) also secreted neutrophil-attracting chemokines in an IL-1-dependent manner *in vitro* (**Fig 2D**). These data suggested that the neutrophil response to OPC is initiated locally in the infected mucosa. While *C*. *albicans* may directly induce chemokine expression in keratinocytes, their production is strongly enhanced by IL-1R signaling.

### G-CSF induces emergency granulopoiesis during OPC

**Fig 3:**
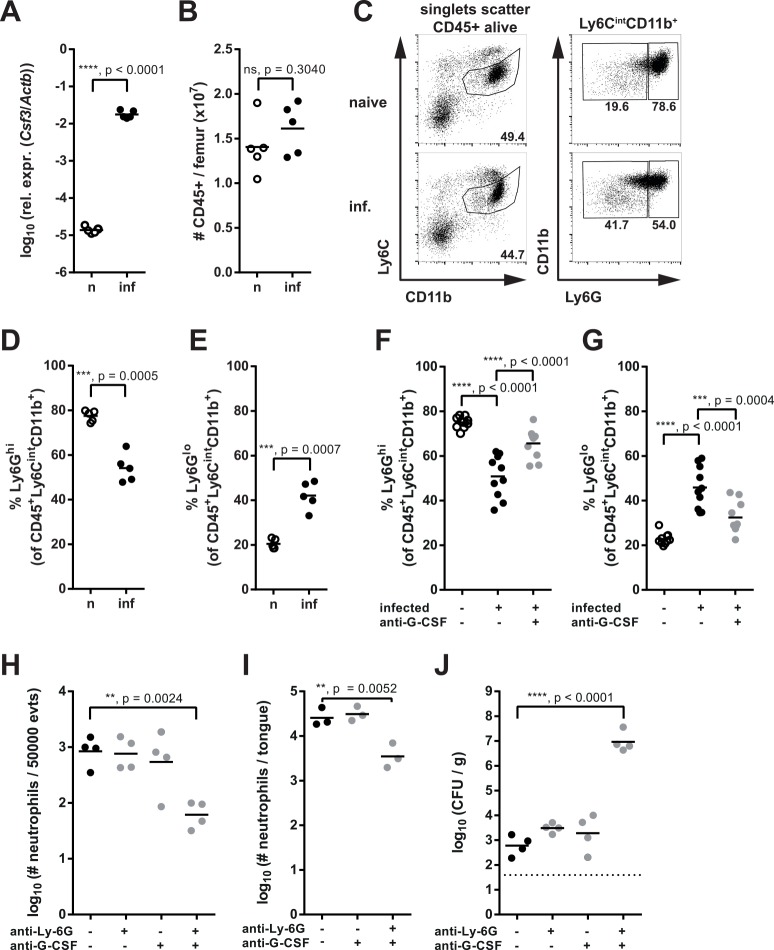
G-CSF induces emergency granulopoiesis during OPC. (**A**) *Csf3* mRNA expression in the tongues of naïve (n) and infected (inf) WT mice was quantified by qRT-PCR 24 hours post-infection. (**B**) Total numbers of CD45^+^ cells in the bone marrow of naïve (n) and infected (inf) WT mice were assessed by flow cytometry. (**C**) Representative FACS plots showing the analysis of Ly6G^hi^ CD11b^+^ Ly6C^int^ mature neutrophils and Ly6G^lo^ CD11b^+^ Ly6C^int^ immature neutrophils in the bone marrow of naïve and infected WT mice. Depicted events were pre-gated on singlets, scatter, CD45^+^ alive cells. Numbers indicate the % of cells in each gate. (**D—E**) Analysis of Ly6G^hi^ mature neutrophils (D) and Ly6G^lo^ immature neutrophils (E) in the bone marrow of naïve (n) and infected (inf) WT mice 24 hours post-infection. (**F–G**) As in D–E, but mice were treated with anti-G-CSF or left untreated prior to infection as indicated. (**H–J**) WT mice were treated with anti-Ly6G and/or anti-G-CSF antibody or left untreated prior to infection as indicated. CD45^+^ CD11b^hi^ Ly6C^int^ neutrophils were quantified in the blood (H) and tongues (I) 24 hours post-infection. The fungal burden in the tongue was determined on day 3 post-infection (J). Each symbol represents an individual mouse and the lines represent the mean (B, D–G) or geometric mean (A, H—J) of each group. Data are pooled from two (F, G) or representative of two independent experiments (A–E, H–J). Statistical analysis was performed using log_10_ transformation (A, H–J) and Student’s t-test with Welch’s correction (A–B, D–E) or a one-way ANOVA with Dunnett’s test (F–J).

In addition to the strong induction of the neutrophil-recruiting chemokines during OPC, the expression of *Csf3*, the gene coding for G-CSF, was also markedly induced in the infected oral mucosa (**Fig 3A**), suggesting that it may contribute to the overall neutrophil response during OPC by boosting granulopoiesis and neutrophil egress from the bone marrow. We therefore analyzed typical surrogate hallmarks of emergency granulopoiesis in the bone marrow. While total numbers of CD45^+^ cells in the bone marrow were unchanged in infected mice compared to naïve controls (**Fig 3B**), we observed a reduction in mature Ly6G^hi^ CD11b^+^ Ly6C^int^ neutrophils (**Fig 3C and 3D**). This response was paralleled by an increase in Ly6G^lo^ CD11b^+^ Ly6C^-^ immature neutrophils (**Fig 3C and 3E**). These reciprocal changes in mature and immature neutrophils in the bone marrow indicated that G-CSF, which was induced by the local infection with *C*. *albicans*, acted at a distance and thereby elicited a systemic response. Indeed, neutralization of G-CSF impaired the induction of emergency granulopoiesis during infection (**Fig 3F and 3G**). The increased demand for neutrophils in the infected tissue was thus compensated by an increased rate of granulopoiesis and mobilization of neutrophils from the bone marrow.

The increased neutrophil output from the bone marrow was relevant for the local antifungal response as indicated by the fact that depletion of the circulating neutrophil pool with an anti-Ly6G-specific antibody (1A8) was not sufficient to blunt the neutrophil response to *C*. *albicans* [6]. Instead, the combination of anti-Ly6G and anti-G-CSF was required for efficient depletion of neutrophils in the blood and in the infected tissue (**Fig 3H and 3I**) resulting in a total loss of fungal control (**Fig 3J**). Note that the reduction of neutrophils by ~1 log (**Fig 3I**) resulted in a ~4-log increase in fungal load over a course of 3 days (**Fig 3J**), while the ~0.5 log reduction in neutrophils in absence of IL-1 signaling (**Fig 1B**) lead to a ~2-log increase in fungal load over the same period of time (**Fig 1C**).

### Endothelial rather than epithelial cells are the primary source of G-CSF production during OPC

**Fig 4:**
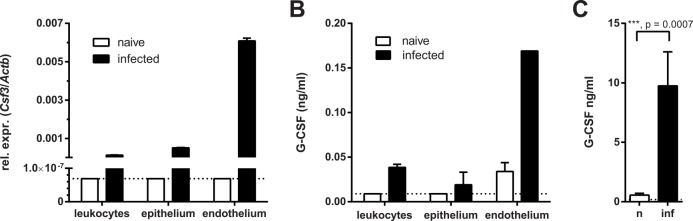
Endothelial cells are the primary source of G-CSF production during OPC. (**A**) CD45^+^ leukocytes, CD45^-^ EpCAM^+^ CD31^-^ epithelial cells, and CD45^-^ EpCAM^-^ CD31^+^ endothelial cells were isolated from the tongues of naïve and infected WT mice by FACS sorting 24 hours post-infection, and *Csf3* mRNA was quantified by qRT-PCR. (**B**) Tongue cell populations were sorted from the tongues of naïve and infected WT mice as in (A). Cell lysates were generated from the sorted populations and G-CSF protein was quantified in the lysates by ELISA. (**C**) G-CSF levels were determined in the serum of naïve (n) and infected (inf) WT mice by ELISA. Data are pooled from two (B) or representative of two (A) or five (C) independent experiments. Bar graphs show the group mean + SD. Statistical analysis was performed using Student’s t-test with Welch’s correction.

Given the prominent role of G-CSF for the overall neutrophil response during OPC we wanted to understand the regulation of this growth factor in more detail. For this, we isolated again CD45^+^ leukocytes, CD45^-^ EpCAM^+^ CD31^-^ keratinocytes, and CD45^-^ EpCAM^-^ CD31^+^ endothelial cells from the tongue of *C*. *albicans*-infected mice and uninfected controls as above (**S3 Fig**) and analyzed *Csf3* transcript levels in each population. Surprisingly, the most prominent expression was observed in the endothelial cell population (**Fig 4A**). Although keratinocytes were previously shown to secrete G-CSF in response to *C*. *albicans in vitro* [26], their contribution *in vivo* was minor (**Fig 4A**). Similarly, leukocytes expressed only very low levels of G-CSF during OPC (**Fig 4A**). To corroborate this unexpected finding, we examined G-CSF protein production by the different cell subsets. Because we were unable to visualize intracellular G-CSF by flow cytometry, we prepared cell lysates from sort-purified tongue cell populations and quantified their G-CSF content by ELISA. Again, by far the highest production of G-CSF was detected in the endothelial cell fraction isolated from infected mice (**Fig 4B**). Further separation of the CD45^-^ EpCAM^-^ CD31^+^ population in podoplanin-positive and podoplanin-negative subsets confirmed that blood endothelial cells rather than lymph endothelial cells were responsible for G-CSF production during OPC (**S4 Fig**). Production of G-CSF by endothelial cells was supported by the observation that high levels of G-CSF could be detected in the serum of infected mice (**Fig 4C**). Together, these data suggested that G-CSF, which is induced in response to local infection with *C*. *albicans*, acts at a distance to promote granulopoiesis and neutrophil mobilization in the bone marrow and thereby sustains a systemic neutrophil response that meets the increased demand of these cells during OPC.

### Endothelial cell-derived G-CSF in the oral mucosa is IL-1 dependent

**Fig 5:**
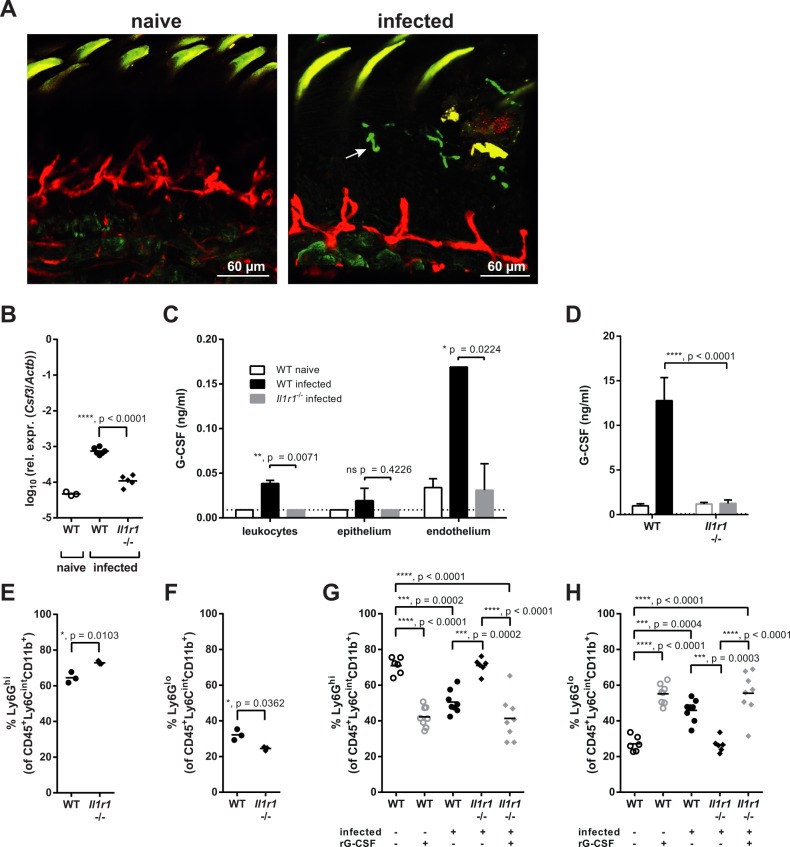
Endothelial cell-derived G-CSF in the oral mucosa is IL-1-dependent. (**A**) VE-cadherin-cre x ROSA26-RFP mice were infected with *C*. *albicans* strain pACT1-GFP or left uninfected. The images show each a 3D reconstruction of a Z stack of a 20x fields of view acquired from whole mount samples of naïve or infected tongues 24 hours post-infection. Fungal hyphae are identified in the avascular tongue epithelium by their morphology and green fluorescence (white arrow). The papillae appear yellow due to both green and red autofluorescence. Muscle cells display green autofluorescence. (**B**) *Csf3* mRNA was quantified in the tongues of naïve WT and infected WT and *Il1r1* ^-/-^ mice by qRT-PCR 24 hours post-infection. (**C**) CD45^+^ leukocytes, CD45^-^ EpCAM^+^ CD31^-^ epithelial cells and CD45^-^ EpCAM^-^ CD31^+^ endothelial cells were isolated from the tongues of naïve WT and infected WT and *Il1r1* ^-/-^ mice by FACS sorting 24 hours post-infection. Cell lysates were prepared from the sorted populations and G-CSF protein was quantified in the lysates by ELISA. The data of the WT mice are identical with those shown in Fig 4B. (**D**) G-CSF levels were determined in the serum of naïve (open bars) and infected (closed bars) WT and *Il1r1* ^-/-^ mice by ELISA 24 hours post-infection. (**E–F**) Quantification of Ly6G^hi^ mature neutrophils (E) and Ly6G^lo^ immature neutrophils (F) in the bone marrow of infected WT and *Il1r1* ^-/-^ mice analyzed 24 hours post-infection. (**G–H**) As in (E–F), but mice were treated with recombinant G-CSF or left untreated as indicated. Each symbol represents an individual mouse and the lines represent the mean (E–H) or geometric mean (B) of each group. Bar graphs in C–D show the group mean + SD. Data are pooled from two (B–C, G–H) or representative of two (A, D–F) independent experiments, with the exception of the naive group in B, which is from one experiment. Statistical analysis was performed using log_10_ transformation (B) and Student’s t-test with Welch’s correction (C, E–F), a one-way ANOVA with Tukey’s test (G—H) or Dunnett’s test (B), or two way ANOVA with Tukey’s test (D).

Next, we investigated how G-CSF secretion by endothelial cells is regulated during OPC. Direct stimulation of endothelial cells by *C*. *albicans* appeared unlikely given their spatial distribution in the oral mucosa. We made use of VE-cadherin-cre x ROSA26-RFP reporter mice [30] to visualize the blood and lymphatic vessels *in situ* and infected them with GFP-expressing *C*. *albicans* (**Fig 5A**). In immunocompetent animals, fungal hyphae were restricted to the avascular tongue epithelium without penetrating the basal epithelial layers. The absence of direct contacts of *C*. *albicans* with endothelial cells suggested that G-CSF production was regulated indirectly.

Given the important role of IL-1 for the overall neutrophil response to *C*. *albicans*, we assessed the IL-1 dependence of G-CSF during OPC. The induction of *Csf3* transcripts was less pronounced in the oral mucosa of *Il1r1* ^-/-^ mice (**Fig 5B**), while basal levels were unchanged (**S2 Fig**). Likewise, G-CSF protein expression by endothelial cells that were sorted from infected tongues was drastically diminished in absence of IL-1R signaling (**Fig 5C**). This translated in strongly diminished G-CSF levels in the serum of *C*. *albicans*-infected *Il1r1* ^-/-^ mice compared to WT controls (**Fig 5D**). In contrast, TNF, which had been proposed to regulate G-CSF expression *in vitro* [31], was not involved in G-CSF production during OPC (**S5 Fig**). As a consequence of the G-CSF defect in *Il1r1* ^-/-^ mice, emergency granulopoiesis was strongly impaired during OPC in these mice, as indicated by the higher ratio of mature to immature neutrophils in the bone marrow of infected *Il1r1* ^-/-^ mice compared to WT controls (**Fig 5E and 5F**). Importantly, *Il1r1* ^-/-^ mice responded to G-CSF treatment, and the administration of recombinant G-CSF was sufficient to fully overcome the defect in emergency granulopoiesis in these mice (**Fig 5G and 5H**).

Together, these data demonstrated, that G-CSF secretion during OPC was controlled by the IL-1 pathway. G-CSF production appeared to underlie a different regulatory mechanism compared to neutrophil chemokine production during OPC given their distinct cellular sources, despite the fact that both, chemokines and G-CSF, depended on IL-1R signaling.

### Keratinocyte-derived IL-1α regulates G-CSF production during OPC

**Fig 6:**
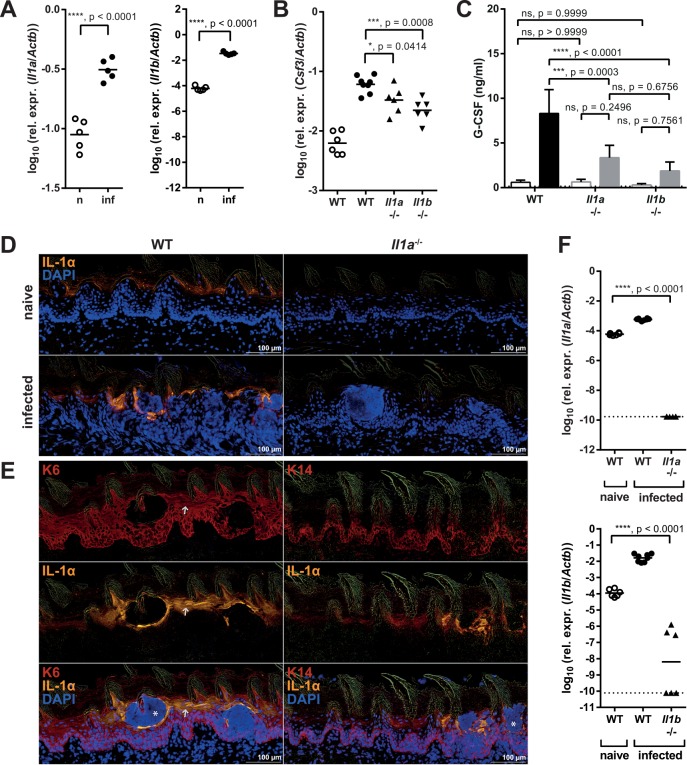
Keratinocyte-derived IL-1α regulates G-CSF production during OPC. (**A**) *Il1a* and *Il1b* mRNA was quantified in the tongues of naïve (n) and infected (inf) WT mice by qRT-PCR 24 hours post-infection. (**B–C**) WT, *Il1a* ^-/-^ and *Il1b* ^-/-^ mice were infected with *C*. *albicans*. *Csf3* mRNA levels in naïve and infected tongue tissue were determined by qRT-PCR (B), and G-CSF serum levels were measured by ELISA (C) before infection (open bars) and 24 hours post-infection (closed bars). (**D**) Immunofluorescent staining of sagittal tongue sections from naïve and infected WT and *Il1a* ^-/-^ mice stained for IL-1α (yellow) and DAPI (blue) 24 hours post-infection. (**E**) Immunofluorescent staining of sagittal tongue sections from infected WT mice stained for keratin-6 (K6, left) or keratin-14 (K14, right) in red, as well as IL-1α (yellow) and DAPI (blue) 24 hours post-infection. Note that the IL-1α signal is absent in neutrophil-rich areas. The white arrow serves as orientation. (**F**) IL-1α and IL-1β mRNA expression in the tongues of naïve WT and infected WT, *Il1a* ^-/-^ and *Il1b* ^-/-^ mice was measured by qRT-PCR 24 hours post-infection. The detection limit, which was calculated using the average Ct (*Actb*) of all samples and Ct (*Il1a*) or Ct (*Il1b*) = 50, is depicted by a dotted line. Each symbol represents an individual mouse (A–B, F) and the lines represent the geometric mean of each group. The bar graph in C shows the group mean + SD. Data are representative of two independent experiments (A, D–E), or pooled from two independent experiments (B–C, F), with the exception of the naïve groups in C, which are the mean + SD of 4 (WT) or 3 (*Il1a* ^-/-^, *Il1b* ^-/-^) animals from one experiment. Statistical analysis was performed using log_10_ transformation (A–B, F) and Student’s t-test with Welch’s correction (A), a one-way ANOVA with Dunnett’s test (B, F) or two-way ANOVA with Tukey’s test (C).

Next, we examined the expression of IL-1α and IL-1β, the two activating ligands of the IL-1R. Both were found strongly induced during OPC (**Fig 6A**). IL-1α and IL-1β contribute to G-CSF production during OPC because the induction of G-CSF expression in the oral mucosa and its release into the serum were strongly impaired in absence of IL-1α or IL-1β (**Fig 6B and 6C**).

IL-1β was expressed predominantly by the hematopoietic compartment as assessed by flow cytometric analysis of intracellular pro-IL-1β (**S6 Fig**). To determine the cellular source of IL-1α in the murine tongue, we applied an immunofluorescence approach, which allowed us to detect IL-1α with high specificity on tissue sections. Basal expression levels observed in the keratinized epithelium of the tongue in naïve mice were strongly enhanced upon infection (**Fig 6D**). Co-staining with antibodies specific for keratinocytes of the tongue (keratin-6) or those of stratum basale (keratin-14) [32] revealed that IL-1α was predominantly produced by differentiated keratinocytes (**Fig 6E**). Note that the IL-1α signal was absent in neutrophil-rich areas, which were identified by DAPI staining.

The availability of preformed IL-1α (but not IL-1β) protein in steady-state (**Fig 6D**, **S6 Fig**), which can be rapidly released in response to stimulation, together with the strategic position of keratinocytes as the first contact point between the host and the infecting fungus, suggested that keratinocyte-derived IL-1α likely functions as an ‘alarmin’ right at the onset of infection to alert the host about fungal invasion and to initiate a protective antifungal response. As such, keratinocyte-derived IL-1α may also act on endothelial cells and thereby contribute to the neutrophil response during OPC.

### G-CSF production by endothelial cells is induced by keratinocyte-derived IL-1α

**Fig 7:**
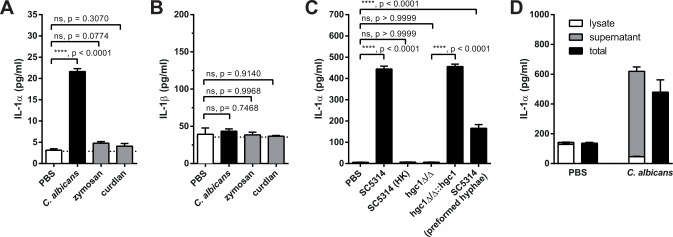
TDKs release IL-1α in response to *C*. *albicans*. (**A—B**) TDKs were stimulated with *C*. *albicans*, zymosan, curdlan or left untreated (PBS), and IL-1α (A) and IL-1β (B) levels were determined in the supernatant by cytometric bead array. (**C**) IL-1α release from TDKs was determined as in (A) after stimulation with live or heat-killed (HK) *C*. *albicans* strain SC5314, with the yeast-locked strain *hgc1*Δ/Δ, its revertant *hgc1*Δ/Δ::*hgc1*, or with preformed hyphae prepared from strain SC5314. (**D**) TDKs were stimulated with *C*. *albicans* or left unstimulated (PBS). Amphotericin B was added after 8 hours of stimulation to prevent hyphal overgrowth. IL-1α levels were determined in the supernatant and in lysates prepared from the cells in the same wells. As a control, triton was added to separate wells containing cells and supernatant to quantify total amounts of IL-1α per well (total). Bar graphs show the group mean + SD. Data are representative of two independent experiments. Statistical analysis was performed using one-way ANOVA with Dunnett’s test (A–B) or Tukey’s test (C).

To delineate the putative crosstalk between keratinocytes and endothelial cells in more detail, we made use of a newly generated cell line of mouse tongue-derived keratinocytes (TDKs) (**S7 Fig**). TDKs expressed high levels of EpCAM and keratin-6 indicating that they represented differentiated oral keratinocytes [32]. Their concurrent expression of keratin-14 was consistent with their origin of basal keratinocytes with stem cell properties. Consistent with published data with keratinocytes from other sources [26], TDKs released IL-1α when stimulated with *C*. *albicans* (**Fig 7A**). Notably, and in contrast to human keratinocytes [28], mouse keratinocytes released no IL-1β (**Fig 7B**). This response was dependent on live and hyphenating fungus because heat-killed or a yeast-locked *C*. *albicans* did not induce IL-1α (**Fig 7C**). Likewise, IL-1α was not induced with zymosan, a yeast cell wall extract, or curdlan, a pure β-glucan preparation (**Fig 7A**). Consistent with our *in vivo* data, the overall amount of IL-1α detected from TDKs in response to live *C*. *albicans* resulted from the release of preformed cytokine and *de novo* biosynthesis of IL-1α (**Fig 7D**).

**Fig 8:**
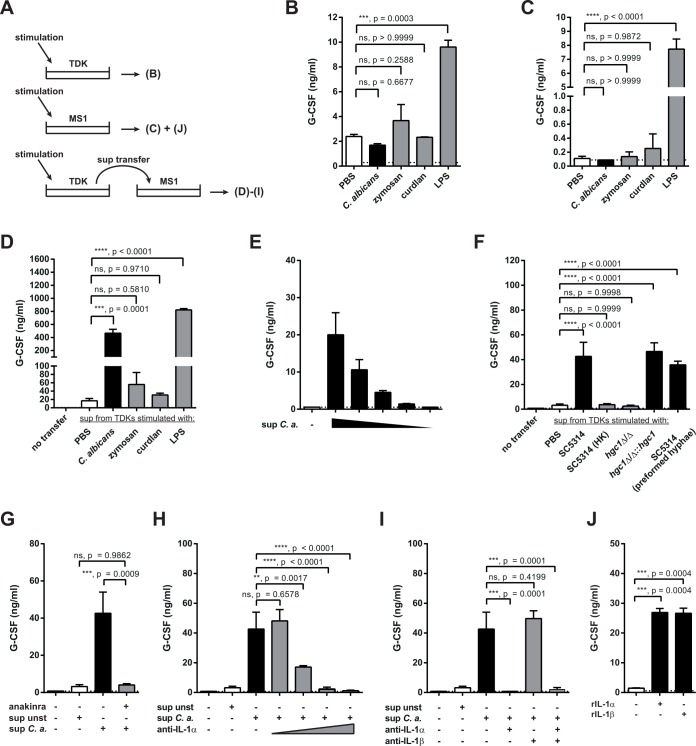
TDK-derived IL-1α induces G-CSF secretion by endothelial cells. (**A**) Schematic overview of the experimental setup used in panels B–J. (**B–J**) G-CSF levels in the supernatants of TDKs (B) and MS1 cells (C–J) were determined by ELISA 24 hours after stimulation. TDKs (B) and MS1 cells (C) were stimulated with *C*. *albicans*, zymosan, curdlan, LPS or left unstimulated (PBS) as indicated. (**D**) MS1 cells were stimulated with the supernatants of stimulated TDKs from (A) (diluted 3-fold in MS1 culture medium). (**E**) MS1 cells were stimulated with the serially diluted supernatant of *C*. *albicans*-stimulated TDKs. (**F**) MS1 cells were stimulated with the TDK supernatants from Fig 7C. (**G**) MS1 cells were pretreated with anakinra as indicated prior to the addition of the supernatant of *C*. *albicans*-stimulated TDKs. (**H—I**) The supernatant of *C*. *albicans*-stimulated TDKs was treated with anti-IL-1α and/or anti-IL-1β as indicated before being transferred to MS1 cells. (**J**) MS1 cells were treated with recombinant IL-1α, IL-1β, or left untreated. The conditions of G-CSF induction shown in (F–I) were measured all in one experiment but are displayed in several individual graphs for better comprehension. Data (B–J) are all representative of at least two independent experiments. Bar graphs show the group mean + SD. Statistical analysis was performed using one-way ANOVA with Dunnett’s test.

Next, we tested the effect of TDKs and TDK-derived IL-1α on endothelial cells for G-CSF induction (**Fig 8A**). For this, we employed an established endothelial cell line, MS1 [33]. TDKs and MS1 cells both did not produce G-CSF when directly stimulated with *C*. *albicans* nor with curdlan or zymosan, although they responded strongly to LPS, which was included as a positive control (**Fig 8B and 8C**). However, MS1 cells secreted high amounts of G-CSF when stimulated with the sterile-filtered supernatant of *C*. *albicans*-stimulated TDKs (**Fig 8D**). This response was dose-dependent (**Fig 8E**) and only observed when TDKs were stimulated with life and hyphenating *C*. *albicans*, but not with heat-killed *C*. *albicans*, a yeast-locked strain of *C*. *albicans* or inert fungal cell wall components such as zymosan and β-glucan (curdlan) (**Fig 8D–8F**). Similar results were obtained when supernatant of freshly isolated mouse oral keratinocytes stimulated with *C*. *albicans* was added to MS1 cells (**S8 Fig**). This indicated that a *C*. *albicans*-induced TDK-derived soluble factor was responsible for G-CSF production by endothelial cells. To test whether this factor was IL-1α, we added anakinra (IL-1R antagonist) or a neutralizing anti-IL-1α antibody into the supernatant-transfer assay. This resulted in a complete abolishment of the response (**Fig 8G–8I**), while adding an anti-IL-1β antibody had no effect on G-CSF induction by TDK-derived factors (**Fig 8I**), consistent with the notion that IL-1β was not produced by murine keratinocytes (**Fig 7B**). IL-1α was not only necessary but also sufficient for triggering G-CSF production in endothelial cells, because MS1 cells secreted large quantities of G-CSF when stimulated with recombinant IL-1α (**Fig 8J**). In summary, these results revealed a novel IL-1α-dependent crosstalk between epithelial and endothelial cells that mediates the induction of G-CSF by *C*. *albicans*.

In conclusion, our data corroborate the notion that the epithelium takes an active part in host defense in barrier tissue through its strategic location and by alerting the immune system about the presence of a pathogenic threat. By demonstrating the relevance of IL-1α release (for G-CSF induction) and chemokine secretion, we provided an example in a physiologically relevant system.

## Discussion

In this study, we describe an essential function of the IL-1 pathway in antifungal immunity in the murine oral mucosa. We demonstrated how IL-1R signaling regulates the neutrophil response against *C*. *albicans* in two ways to prevent fungal growth and dissemination. Specifically, it promotes the production of chemokines by oral keratinocytes for neutrophil recruitment from the circulating pool, and it induces G-CSF secretion from endothelial cells to enhance granulopoiesis in the bone marrow to meet the rapid demand for neutrophils in the tissue. IL-1R signaling thereby translates the local response to a tissue-specific infection into a systemic response. The availability of preformed IL-1α from keratinocytes, which are the first cells to be exposed to *C*. *albicans* during infection, is critical for the initiation of the response. Together, our data demonstrate how signaling through the IL-1R coordinates a cellular crosstalk between keratinocytes, endothelial cells and neutrophils for optimal control of *C*. *albicans* in the oral mucosa.

Rapid infiltration of neutrophils to the site of infection is a hallmark of the inflammatory response to OPC and critical for the confinement of the fungus in the mucosal epithelium [6]. The neutrophil response in the oral mucosa was originally thought to be controlled by the IL-17 pathway, which itself is highly critical for fungal control during OPC [14]. However, previous work from our laboratory showed that the key protective function of IL-17 is uncoupled from the neutrophil response [6]. Although IL-17 signaling can enhance the expression of neutrophil chemokines and granulopoietic factors [14], IL-17 is not required for neutrophil chemotaxis and function during OPC [6]. Instead, neutrophil trafficking to the oral mucosa during acute infection is under the control of IL-1R signaling as our data demonstrate. IL-1R deficiency is associated with impaired neutrophil recruitment and defective fungal control in response to OPC. Our data are consistent with previous reports demonstrating a role for IL-1R signaling in antifungal defense in different settings including systemic candidiasis [24], a model of mixed oral and systemic candidiasis [34], *A*. *fumigatus* keratitis [35] and invasive pulmonary aspergillosis [36]. The mechanism of IL-1R-mediated protection, however, was not addressed in most of these studies. Here, we used the model of OPC to dissect the impact of IL-1R signaling on neutrophil mobilization and recruitment in response to *C*. *albicans* infection at a cellular and molecular level.

Keratinocytes take center stage in the coordination of the IL-1-mediated neutrophil response during OPC. They act as the major producers of neutrophil-recruiting chemokines. Chemokine production by keratinocytes was greatly enhanced by IL-1R signaling and at least in part through the autocrine activity of IL-1α. A similar mechanism for enhanced chemokine secretion by keratinocytes was described before in the context of *Staphylococcus aureus* skin infection [37]. A second mechanism, by which keratinocytes promote the neutrophil response during OPC, is by the induction of G-CSF via their capacity to produce IL-1α. Our data thus link IL-1 signaling and G-CSF production. G-CSF regulates granulopoiesis and neutrophil mobilization in the bone marrow, which is critical to meet the rapidly increasing demand of neutrophils in the infected tissue. Administration of recombinant G-CSF in mice is sufficient to drive emergency granulopoiesis as we and others have shown [38], and recombinant G-CSF is widely used therapeutically to treat neutropenia [39]. G-CSF has also been used successfully in the treatment of chronic mucocutaneous candidiasis [40]. Besides its host-beneficial role in hematopoiesis and protection from infection, dysregulated production of G-CSF has been linked to autoinflammatory disorders such as psoriasis and inflammatory arthritis [41–43] and targeting G-CSF has been proposed as a therapeutic approach against these diseases [44].

We identified endothelial cells in the oral tissue to be the major producers of G-CSF during OPC, while we were unable to detect G-CSF expression by murine keratinocytes in response to *C*. *albicans*. Direct secretion of G-CSF into the bloodstream by endothelial cells facilitates its delivery to the distant bone marrow and the induction of a systemic response. During systemic bacterial infections, G-CSF production by the endothelium was shown to be a direct and TLR4-dependent response of the vasculature to endotoxin stimulation which lead to the induction of emergency granulopoiesis [38]. During OPC, we found no evidence for direct response of the endothelium to *C*. *albicans*, and endothelial cells were not activated by the fungus *in vitro*. Instead, G-CSF was produced as a result of an IL-1-dependent crosstalk between keratinocytes and endothelial cells. IL-1α was critical for the secretion of G-CSF into the circulation during OPC, and it was sufficient for stimulating G-CSF production by endothelial cells in culture, consistent with a published report [31]. In contrast, TNF, which was also reported to induce G-CSF production by endothelial cells *in vitro* [31], did not regulate G-CSF production during OPC.

In addition to IL-1α, IL-1β also contributes to the antifungal response *in vivo* and at least partially compensates in absence of IL-1α. IL-1β was also induced in the oral mucosa during infection. Consistent with the notion that murine keratinocytes are unable to produce IL-1β [28], we found this cytokine to be expressed by the hematopoietic compartment. IL-1β induction in response to *C*. *albicans* was shown previously to depend on the NLRP3 inflammasome in different infection models [21,34,45] and NLRP3 was required in the hematopoietic compartment [29]. In addition to the NLRP3 inflammasome, the NLRC4 inflammasome was also shown to contribute to protection from OPC [29]. Whether its function at the level of the mucosal stroma is linked to the IL-1 pathway and whether it is involved in IL-1α production in keratinocytes was not addressed.

We found that the overall IL-1α response resulted from the release of preformed and intracellularly stored IL-1α on the one hand, and from the induction of *de novo* synthesis on the other hand. The release of IL-1α from keratinocytes correlates with the induction of cell damage [46], suggesting that IL-1α release may be a consequence of cell death induced by fungal invasion. It remains to be determined whether IL-1α secretion by oral keratinocytes in response to *C*. *albicans* infection depends on caspase-1 as was shown to be the case for bone marrow-derived MNPs differentiated with GM-CSF [20]. The dependence of IL-1α secretion on life and filamenting fungus is consistent with the implication of candidalysin in this process. This fungal peptide toxin has recently been identified as a critical virulence factor promoting epithelial cell damage and the release of cytoplasmic molecules, some of which may act as alarmins [47]. Importantly, our data demonstrate the biological relevance of keratinocyte-derived factors induced by *C*. *albicans* for the induction of inflammation and protection from infection *in vivo*, and they dissect the mechanism how IL-1 coordinates the neutrophil response against the fungus in the oral epithelium.

## Materials and Methods

### Ethics statement

All mouse experiments described in this study were conducted in strict accordance with the guidelines of the Swiss Animal Protection Law and were performed under protocols approved by the Veterinary office of the Canton Zurich, Switzerland (license number 201/2012 and 183/2015). All efforts were made to minimize suffering and ensure the highest ethical and humane standards.

### Mice and depletion strategies

WT C57BL/6J mice were purchased from Janvier Elevage. *Il1a* ^-/-^ mice (a kind gift from Manfred Kopf) [48], *Il1b* ^-/-^ mice [48] (a kind gift from Manfred Kopf and Hans-Dietmar Beer) and *Il1r1* ^-/-^ mice [49] were bred at the Laboratory Animal Service Center (University of Zürich, Switzerland). VE-cadherin-cre x ROSA26-RFP reporter mice [30] and *Tnf* ^-/-^ mice (a kind gift from Annette Oxenius) [50] were bred at the Rodent Center HCI at ETH Zürich. All mice were on the C57BL/6 background, kept in specific pathogen-free conditions and used at 6–15 weeks of age. For neutrophil depletion, mice were treated with anti-Ly6G (clone 1A8, BioXCell, 150μg per mouse i.p. on day -1) and/or anti-G-CSF (clone 67604, R&D Systems, 10μg per mouse per day i.p. starting from day -1), as indicated. For inhibition of emergency granulopoiesis, mice were treated with anti-G-CSF (10μg per mouse per day i.p. on day -1 and day 0). For induction of emergency granulopoiesis, mice were injected with human recombinant G-CSF (Filgrastim, Amgen, 5 μg/mouse i.p. at 5h and 17h post-infection).

### Fungal strain and infections

The *C*. *albicans* strain SC5314 was used for all experiments except where stated otherwise. The yeast-locked strain *hgc1*Δ/Δ and its revertant *hgc1*Δ/Δ:HGC1 [51] were obtained from N. Gow (Aberdeen). The strain pACT1-GFP [52] was obtained from C. Reis e Sousa. All strains were grown in YPD medium at 30°C for 15–18 hours. Mice were infected with 2.5x10^6^ cfu *C*. *albicans* sublingually as described [53] without immunosuppression. Mice were monitored for morbidity and euthanized in case they showed severe signs of pain or distress. For determination of fungal burden, the tongue of euthanized animals was removed, homogenized in sterile 0.05% NP40 in H_2_O for 3 minutes at 25 Hz using a Tissue Lyzer (Qiagen) and serial dilutions were plated on YPD agar containing 100 μg/ml ampicillin. The detection limit corresponds to one colony divided by the mean weight of the tongues in the experiment. For heat-killing 10^8^ yeast cells were boiled for 45 minutes. Preformed hyphae were generated in keratinocyte medium at 37°C, 5% CO_2_ for 24 hours.

### Isolation of tongue and bone marrow cells

Mice were anaesthetized with a sublethal dose of Ketamine (100 mg/kg), Xylazin (20 mg/kg) and Acepromazin (2.9 mg/kg), and perfused by injection of PBS into the right heart ventricle prior to removing the tongue and/or the bones. Tongues were cut into fine pieces and digested with DNase I (200 μg/ml, Roche) and Collagenase IV (4.8 mg/ml, Invitrogen) in PBS at 37°C for 45–60 minutes. Single cell suspensions were passed through a 70 μm strainer using ice-cold PBS supplemented with 1% FCS and 2 mM EDTA and analyzed by flow cytometry (see below). For intracellular cytokine staining, tongue leukocytes were enriched over a 40% percoll gradient before antibody staining and analysis. Bone marrow was flushed from femurs using PBS and passed through a 70 μm strainer using ice-cold PBS supplemented with 1% FCS and 2mM EDTA. Erythrocytes were lyzed using erythrocyte lysis buffer (0.3 M NH_4_Cl, 28 μM NaHCO_3_, 125 μM EDTA), and leukocytes were analyzed by flow cytometry.

### Cell lines

Tongue-derived keratinocytes (TDKs) were obtained from WT mice. The tongue was cut to obtain ventral and dorsal parts. The dorsal part of the tongue was freed from muscle tissue with a scalpel and floated on DMEM (PAA) containing 0.8% trypsin (Thermo Fischer) for 40 minutes with the epithelial side facing upwards. After incubation, the epithelial tissue was separated from the lamina propria, and keratinocytes were isolated by incubating in DMEM medium containing DNase I (Roche, 200 μg/ml) for 30 minutes and occasional vortexing and filtering through a 70 μm strainer. The cells were pelleted, resuspended in keratinocyte medium (S1 Table, adapted from [54]) and cultivated in cell culture plates coated with collagen (Bornstein and Traub Type IV, Sigma Aldrich, 25μg/ml). The blood endothelial cell line MS1 [33] was kept in DMEM medium supplemented with 5% FCS and Penicillin/Streptomycin. Prior to stimulation experiments, TDK and MS1 cells were rested for 48 hours and then stimulated with recombinant IL-1α (Peprotech, 20 ng/ml), IL-1β (Peprotech, 20 ng/ml), anakinra (250 μg/ml), zymosan (20 μg/ml), curdlan (200 μg/ml), LPS (100 ng/ml) or *C*. *albicans* at MOI = 3 for 24 hours. Amphotericin B was added 8 hours post-stimulation where stated explicitly. Supernatants were collected for analysis. In some cases, cell lysates were generated by adding 0.1% Triton-X 100 in PBS to the supernatant-free cells. To determine the total amount of IL-1α in cells and supernatant, Triton-X 100 was added to separate culture wells at a final concentration of 0.1%. For supernatant transfer experiments, TDKs were stimulated for 24 hours as described, supernatants were removed, sterile-filtered and added to MS1 cells at a 1:3 dilution. G-CSF production by MS1 cells was determined by ELISA after 24 hours (see below). In some experiments, TDK supernatants were treated with anti-IL-1α (clone ALF-161, BioXCell, 20 μg/ml) and/or anti-IL-1β (clone B122, BioXCell, 20 μg/ml) before they were added to MS1 cells, or MS1 cells were pretreated with anakinra for 30 minutes before addition of TDK supernatant.

### Flow cytometry

All antibodies were from BioLegend, if not stated otherwise. For flow cytometry analyses of neutrophils, single cell suspensions of tongues, bone marrow or blood were stained in ice-cold PBS supplemented with 1% FCS, 5 mM EDTA, and 0.02% NaN_3_ with LIVE/DEAD Fixable Near-IR Stain (Life Technologies), anti-CD45.2 (clone 104), anti-CD11b (clone M1/70), anti-Ly6C (clone AL-21, BD Biosciences) and anti-Ly6G (clone 1A8). For flow cytometry analyses of tongue keratinocytes and endothelial cells, single cell suspensions were stained in ice-cold PBS supplemented with 1% FCS, 5 mM EDTA, and 0.02% NaN_3,_ with LIVE/DEAD Fixable Near-IR Stain or 7AAD (BD Pharmingen), anti-CD45.2 (clone 104), anti-EpCAM (clone G8.8) and anti-CD31 (clone MEC13.3). In some experiments, anti-podoplanin (clone 8.1.1) was included. For intracellular cytokine staining, tongue cells were first incubated in LIVE/DEAD Fixable Near-IR Stain and surface marker antibodies. After fixation and permeabilization using BD Cytofix/Cytoperm (BD Biosciences) the cells were incubated in Perm/Wash buffer (BD Biosciences) containing anti-pro-IL-1β (clone NJTEN3) or the respective isotype control antibody. Cells were acquired on a FACS LSRII (BD Biosciences) or on a FACS Gallios (Becton Coulter), and data were analyzed with FlowJo software (Tristar). For all experiments, the data were pre-gated on live single cells. For isolating tongue cell subsets by FACS sorting, single cell suspensions of five tongues were pooled, stained as described above, and sorted on a FACS AriaII (BD Biosciences) using FCS as a collection medium.

### RNA isolation and quantitative RT-PCR

Isolation of total RNA from bulk tongues or sorted cell populations was carried out according to standard protocols using Trizol Reagent (Sigma) or Trizol LS Reagent (Life Technologies). cDNA was generated by RevertAid (Thermo Scientific). Quantitative PCR was performed using SYBR Green (Roche) and a Rotor-Gene 3000 (Corbett Research) or a QuantStudio 7 Flex (LifeTechnologies). The primers were *Actb* fwd 5'-CCCTGAAGTACCCCATTGAAC-3', *Actb* rev 5'-CTTTTCACGGTTGGCCTTAG-3'; *Cxcl1* fwd 5'-CCGCTCGCTTTCTGTG-3', *Cxcl1* rev 5'-GCAGCTCATTGGCGATAG-3'; *Cxcl2* fwd 5'-AGTGAACTGCGCTGTCAATGC-3', *Cxcl2* rev 5'-GCAAACTTTTTGACCGCCCT-3'; *Cxcl5* fwd 5'- GAAAGCTAAGCGGAATGCAC-3', *Cxcl5* rev 5'-GGGACAATGGTTTCCCTTTT-3'; *Csf3* fwd 5'-CTTAAGTCCCTGGAGCAAGTG-3', *Csf3* rev GTGGCCCAGCAACACCAG; *Il1a* fwd 5'-GGGAAGATTCTGAAGAAGAG-3', *Il1a* rev 5'-TAACAGGATATTTAGAGTCG-3'; *Il1b* fwd 5'-TACAGGCTCCGAGATGAACA-3', *Il1b* rev 5'-AGGCCACAGGTATTTTGTCG-3'. All qPCR assays were performed in duplicate and the relative gene expression (rel. expr.) of each gene was determined after normalization with β-actin transcript levels.

### Preparation of cell lysates from sorted cell populations

For the preparation of cell lysates from sorted tongue cell populations, Brefeldin A (250 μg / mouse) was injected i.p. 6 hours before removal of the tongue tissue. Brefeldin A was also added during the antibody staining and supplemented in the collection medium during cell sorting. Sorted cells were washed with PBS, and 400’000 cells of each population (60’000 for naïve leukocytes, 25’000 for LECs) were lysed in 100 μl 0.1% Triton-X 100 in PBS containing cOmplete protease inhibitor cocktail (Roche) using a Tissue Lyzer (Qiagen) for 3 minutes at 25 Hz. The G-CSF content of the lysates was analyzed immediately by ELISA (see below).

### Protein analysis by ELISA and CBA

G-CSF protein in the serum, in cell lysates or in cell culture supernatants was determined by sandwich ELISA using purified anti-G-CSF (clone 67604, R&D Systems) for coating and biotinylated polyclonal rabbit anti-G-CSF (Peprotech) for detection according to standard protocols. For determination of IL-1α and IL-1β levels in cell culture supernatants, cytometric bead array assays (BD Biosciences) were performed according to the manufacturer’s instructions. The detection limits are indicated by a dotted line.

### Immunofluorescence and whole mount confocal microscopy

Tongues were embedded in OCT (Sakura), snap-frozen in liquid nitrogen and stored at -20°C. For immunofluorescence staining, sagittal cryosections (9 μm), were fixed with acetone for 10 minutes at room temperature and stained with anti-IL-1α (clone ALF-161, BioXCell), anti-K6 (BioLegend, poly19057), anti-K14 (BioLegend, poly19053) and 4′,6’-Diamidino-2-phenylindole dihydrochloride (DAPI, Sigma-Aldrich). Slides were mounted with Mowiol (VWR International AG), scanned with a NanoZoomer 2.0 HT (Hamamatsu Photonics K.K.) using 10x and 20x objectives, and analyzed using NDP.scan 2.5.88. All scale bars indicate 100 μm.

For whole mount confocal microscopy, tongues were longitudinally cut with a scalpel and fresh tissue was placed on 35mm microscopy dishes (IBIDI) with PBS. Z stack tile scans were acquired with an SP8 confocal microscope (Leica) equipped with a 20x APO objective (0.7 N.A.) covering large areas of the tongue tissue. 3D maximum projections and mosaic reconstructions of the acquired data were generated with IMARIS (Bitplane).

### Statistics

Statistical significance was determined by Student’s t-test with Welch’s correction, one-way ANOVA with Dunnett’s or Tukey’s multiple comparison test, or two-way ANOVA with Tukey’s multiple comparison test where appropriate using GraphPad Prism software with *, p < 0.05; **, p < 0.01; ***, p < 0.001; **** p < 0.0001. For data plotted on a logarithmic scale the geometric mean is indicated, and data were log-transformed before statistical analysis.

## Supporting Information

S1 Fig Flow cytometry analysis of CD45^+^ Ly6C^int^ Ly6G^+^ neutrophils in the tongues of OPC infected mice. WT mice were infected sublingually with *C*. *albicans*. FACS plots show the gating strategy used for the quantification of CD45^+^ Ly6C^int^ Ly6G^+^ neutrophils 24 hours post-infection. Data shown were pre-gated on scatter and single cells. Numbers indicate % of cells in each population.
(PDF)

S2 Fig Basal expression of IL-1R-dependent genes. *Cxcl1*, *Cxcl2*, *Cxcl5* and *Csf3* expression was quantified by qRT-PCR in the tongue of naïve WT and *Il1r1*-/- mice. Each symbol represents an individual mouse and the lines represent the geometric mean of each group. Statistical analysis was performed using log_10_ transformation and Student’s t-test with Welch’s correction.
(PDF)

S3 Fig Isolation of CD45^+^ leukocytes, CD45^-^ EpCAM^+^ CD31^-^ keratinocytes and CD45^-^ EpCAM^-^ CD31^+^ endothelial cells from the tongues of OPC infected mice. WT mice were infected sublingually with *C*. *albicans*. FACS plots show the gating strategy used for the isolation of CD45^+^ leukocytes, CD45- EpCAM^+^ CD31^-^ epithelial cells, and CD45^-^ EpCAM^-^ CD31^+^ endothelial cells 24 hours post-infection. Data shown were pre-gated on scatter and single cells. Numbers indicate % of cells in each population.
(PDF)

S4 Fig G-CSF is produced by blood endothelial cells rather than lymph endothelial cells. WT mice were infected sublingually with *C*. *albicans* and CD45^-^ EpCAM^+^ epithelial cells, CD45^-^ EpCAM^-^ CD31^+^ podoplanin^-^ blood endothelial cells and CD45^-^ EpCAM^-^ CD31^+^ podoplanin^+^ lymph endothelial cells were isolated from the tongues 24 hours post-infection. (**A**) FACS plots show the gating strategy used for the isolation of the cell populations. (**B**) G-CSF protein was quantified in the lysates of the sorted populations by ELISA. Data are representative of two independent experiments.
(PDF)

S5 Fig TNF does not regulate G-CSF production during OPC. G-CSF levels were determined in the serum of infected WT and *Tnf* ^-/-^ mice by ELISA 24 hours post-infection. Bar graphs show the group mean + SD. Statistical analysis was performed using log_10_ transformation and Student’s t-test with Welch’s correction.
(PDF)

S6 Fig IL-1β is expressed by the hematopoietic compartment. pro-IL-1β expression was assessed in naïve an infected WT mice by intracellular staining and flow cytometry at 24 hours post-infection. Data show the comparison between naïve and infected animals (**A**) and between isotype and anti-pro-IL-1β antibody staining (**B**). Cells were pre-gated on scatter and single cells. Numbers indicate % of cells in each population. Data are representative of two independent experiments.
(PDF)

S7 Fig Characterization of the murine tongue-derived keratinocyte (TDK) cell line. **(A)** Histogram shows EpCAM staining of TDKs (filled histogram). MS1 endothelial cells were included as a negative control (open histogram). Cells were gated on singlets and scatter. **(B)** Keratin-6 (K6, in red, left column) and keratin-14 (K14, in red, right column) staining of TDKs by immunofluorescence. MS1 endothelial cells were included as a negative control (bottom). Nuclei were labelled with DAPI (blue).
(PDF)

S8 Fig Primary mouse oral keratinocytes also release G-CSF-inducing factor(s) in response to *C*. *albicans*. The supernatants of primary tongue-derived keratinocytes that were stimulated with *C*. *albicans*, zymosan, LPS or left unstimulated (PBS) as indicated were transferred to unstimulated MS1 cells (diluted 3-fold in MS1 culture medium). G-CSF levels in the supernatants of MS1 cells were determined by ELISA 24 hours after transfer.
(PDF)

S1 Table Keratinocyte medium (adapted from [54]). (PDF)
