# Supplementary figures and images for: IL-1 Coordinates the Neutrophil Response to C. albicans in the Oral Mucosa
Source: PLoS Pathog. 2016 Sep 15;12(9):e1005882. doi: 10.1371/journal.ppat.1005882 (PMC5025078; doi:10.1371/journal.ppat.1005882)

FigS1

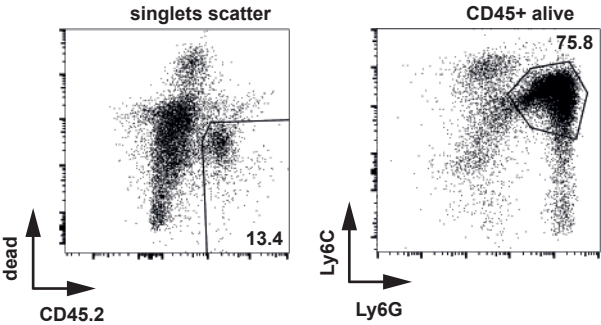

Supplement: S1 Fig — WT mice were infected sublingually with C. albicans. FACS plots show the gating strategy used for the quantification of CD45+ Ly6Cint Ly6G+ neutrophils 24 hours post-infection. Data shown were pre-gated on scatter and single cells. Numbers indicate % of cells in each population. (PDF) [file ppat.1005882.s001.pdf]

FigS2

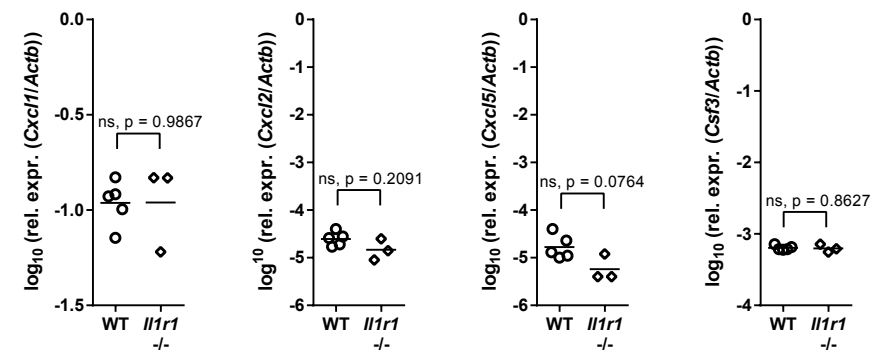

Supplement: S2 Fig — Cxcl1, Cxcl2, Cxcl5 and Csf3 expression was quantified by qRT-PCR in the tongue of naïve WT and Il1r1-/- mice. Each symbol represents an individual mouse and the lines represent the geometric mean of each group. Statistical analysis was performed using log10 transformation and Student’s t-test with Welch’s correction. (PDF) [file ppat.1005882.s002.pdf]

FigS3

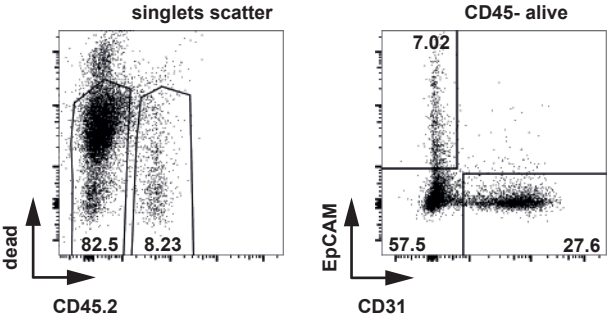

Supplement: S3 Fig — WT mice were infected sublingually with C. albicans. FACS plots show the gating strategy used for the isolation of CD45+ leukocytes, CD45- EpCAM+ CD31- epithelial cells, and CD45- EpCAM- CD31+ endothelial cells 24 hours post-infection. Data shown were pre-gated on scatter and single cells. Numbers indicate % of cells in each population. (PDF) [file ppat.1005882.s003.pdf]

FigS4

A

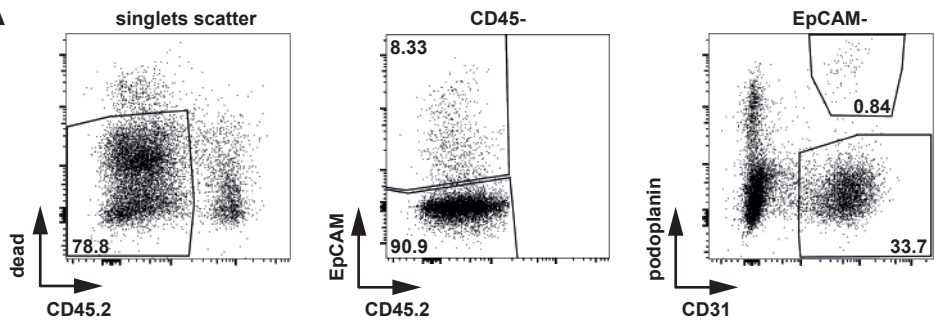

B

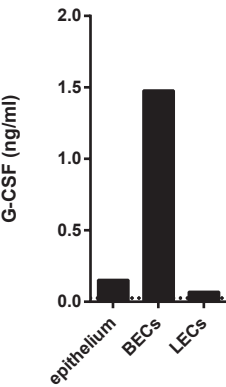

Supplement: S4 Fig — WT mice were infected sublingually with C. albicans and CD45- EpCAM+ epithelial cells, CD45- EpCAM- CD31+ podoplanin- blood endothelial cells and CD45- EpCAM- CD31+ podoplanin+ lymph endothelial cells were isolated from the tongues 24 hours post-infection. (A) FACS plots show the gating strategy used for the isolation of the cell populations. (B) G-CSF protein was quantified in the lysates of the sorted populations by ELISA. Data are representative of two independent experiments. (PDF) [file ppat.1005882.s004.pdf]

FigS5

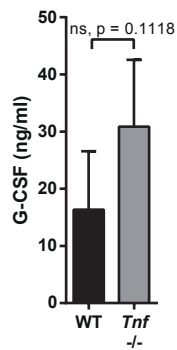

Supplement: S5 Fig — G-CSF levels were determined in the serum of infected WT and Tnf -/- mice by ELISA 24 hours post-infection. Bar graphs show the group mean + SD. Statistical analysis was performed using log10 transformation and Student’s t-test with Welch’s correction. (PDF) [file ppat.1005882.s005.pdf]

FigS6

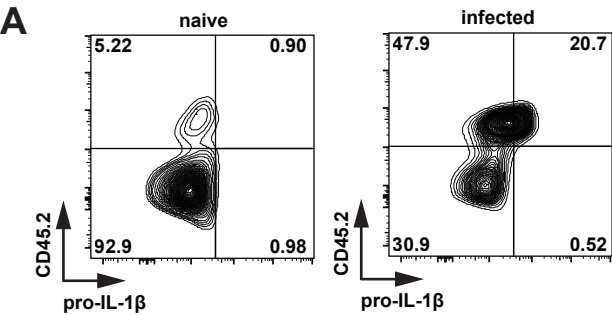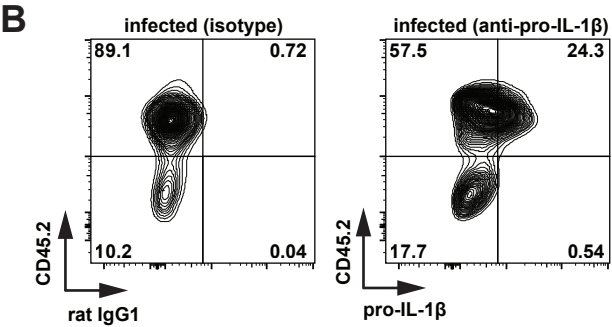

Supplement: S6 Fig — pro-IL-1β expression was assessed in naïve an infected WT mice by intracellular staining and flow cytometry at 24 hours post-infection. Data show the comparison between naïve and infected animals (A) and between isotype and anti-pro-IL-1β antibody staining (B). Cells were pre-gated on scatter and single cells. Numbers indicate % of cells in each population. Data are representative of two independent experiments. (PDF) [file ppat.1005882.s006.pdf]

**FigS7**

**A**

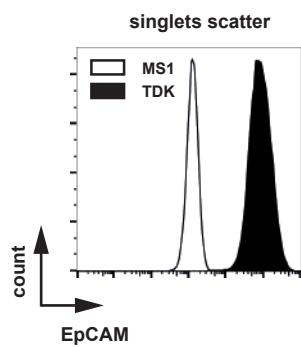

**B**

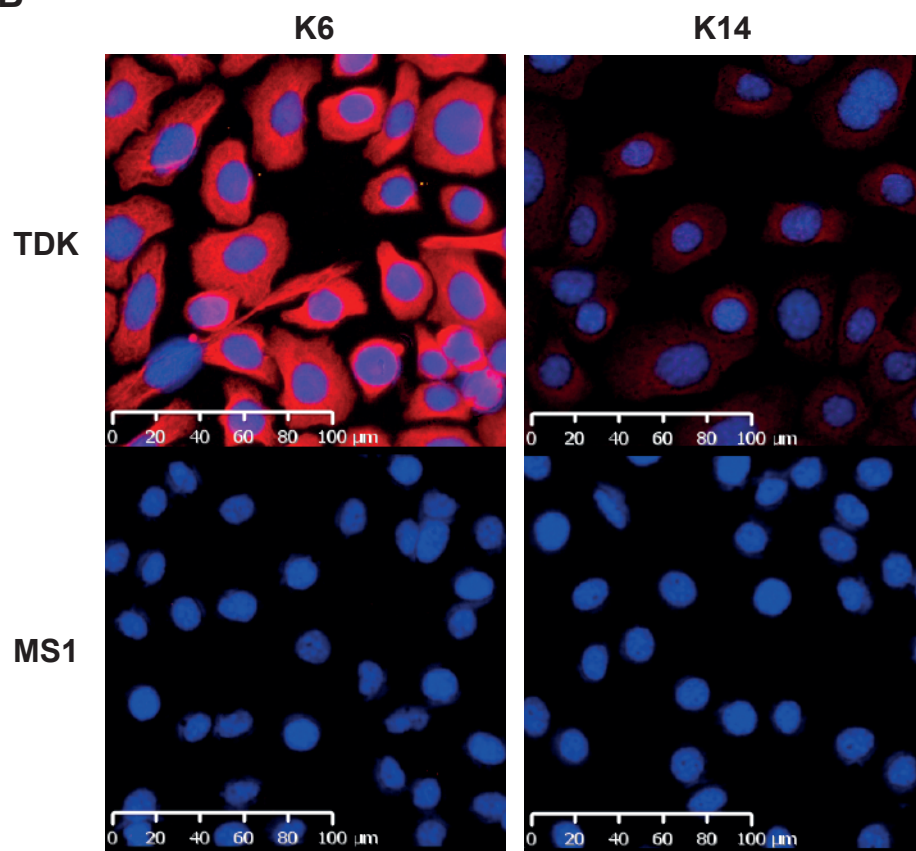

Supplement: S7 Fig — (A) Histogram shows EpCAM staining of TDKs (filled histogram). MS1 endothelial cells were included as a negative control (open histogram). Cells were gated on singlets and scatter. (B) Keratin-6 (K6, in red, left column) and keratin-14 (K14, in red, right column) staining of TDKs by immunofluorescence. MS1 endothelial cells were included as a negative control (bottom). Nuclei were labelled with DAPI (blue). (PDF) [file ppat.1005882.s007.pdf]

FigS8

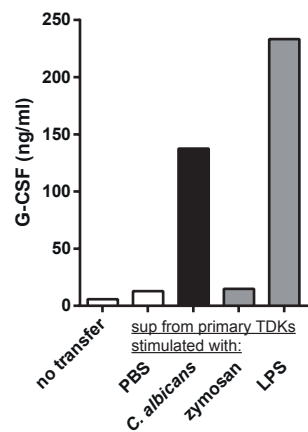

Supplement: S8 Fig — The supernatants of primary tongue-derived keratinocytes that were stimulated with C. albicans, zymosan, LPS or left unstimulated (PBS) as indicated were transferred to unstimulated MS1 cells (diluted 3-fold in MS1 culture medium). G-CSF levels in the supernatants of MS1 cells were determined by ELISA 24 hours after transfer. (PDF) [file ppat.1005882.s008.pdf]
