# Supplementary material for: IL-1 Coordinates the Neutrophil Response to C. albicans in the Oral Mucosa
Source: PLoS Pathog. 2016 Sep 15;12(9):e1005882. doi: 10.1371/journal.ppat.1005882 (PMC5025078; doi:10.1371/journal.ppat.1005882)
Supplement: S1 Table — (PDF) [file ppat.1005882.s009.pdf]

S1 Table

keratinocyte medium

KGF medium		1 part
dF medium		2 parts
EGF	Sigma #E4127	10 ng/ml

KGF medium

	company cat. #	$conc_{final}$
MEM Eagle	Sigma M8167	
insulin	Sigma I5500	5 µg/ml
transferrin	Sigma T8158	10 µg/ml
phosphoethanolamine	Sigma P0503	1.4 µg/ml
ethanolamine	Sigma E0135	0.1%
hydrocortisone	Calbiochem 386698	360 ng/ml
$CaCl_2$ dihydrate (M = 147 g/mol)	Merck	6.6 µg / ml (45 µM)
L-glutamine	Invitrogen	2 mM
penicillin and streptomycin	Invitrogen	100 IU/ml and 100 µg/ml
Ca2+-free FCS	PAA BioRad 142-2842	8%

dF medium

	company cat. #	$conc_{final}$
defined kerationcyte-SFM	Life Technologies 10744-019	
penicillin and streptomycin	Invitrogen	100 IU/ml and 100 µg/ml
cholera toxin	Sigma C-8052	10^{10}
